# Excess All-Cause Mortality in China After Ending the Zero COVID Policy

**DOI:** 10.1001/jamanetworkopen.2023.30877

**Published:** 2023-08-24

**Authors:** Hong Xiao, Zhicheng Wang, Fang Liu, Joseph M. Unger

**Affiliations:** 1 Public Health Sciences Division, Fred Hutchinson Cancer Research Center, Seattle, Washington; 2 Independent researcher

## Abstract

**Importance:**

In China, the implementation of stringent mitigation measures kept COVID-19 incidence and excess mortality low during the first years of the pandemic. However, China’s decision to end its dynamic zero COVID policy (a proactive strategy that deploys mass testing and strict quarantine measures to stamp out any outbreak before it can spread) in December 2022 resulted in a surge in COVID-19 incidence and hospitalizations. Despite worldwide attention given to this event, the actual impact of this sudden shift in policy on population mortality has not been empirically estimated.

**Objective:**

To assess the association of the sudden shift in China’s dynamic zero COVID policy with mortality using empirical and syndromic surveillance data.

**Design, Setting, and Participants:**

This cohort study analyzed published obituary data from 3 universities in China (2 in Beijing and 1 in Heilongjiang) and search engine data from the Baidu index (BI; weighted frequency of unique searches for a given keyword relative to the total search volume on the Baidu search engine) in each region of China from January 1, 2016, to January 31, 2023. Using an interrupted time-series design, analyses estimated the relative change in mortality among individuals 30 years and older in the universities and the change in BI for mortality-related terms in each region of China from December 2022 to January 2023. Analysis revealed a strong correlation between Baidu searches for mortality-related keywords and actual mortality burden. Using this correlation, the relative increase in mortality in Beijing and Heilongjiang was extrapolated to the rest of China, and region-specific excess mortality was calculated by multiplying the proportional increase in mortality by the number of expected deaths. Data analysis was performed from February 10, 2023, to March 5, 2023.

**Exposure:**

The end to the dynamic zero COVID policy in December 2022 in China.

**Main Outcomes and Measures:**

Monthly all-cause mortality by region.

**Results:**

An estimated 1.87 million (95% CI, 0.71 million-4.43 million; 1.33 per 1000 population) excess deaths occurred among individuals 30 years and older in China during the first 2 months after the end of the zero COVID policy. Excess deaths predominantly occurred among older individuals and were observed across all provinces in mainland China except Tibet.

**Conclusions and Relevance:**

In this cohort study of the population in China, the sudden lifting of the zero COVID policy was associated with significant increases in all-cause mortality. These findings provide valuable insights for policy makers and public health experts and are important for understanding how the sudden propagation of COVID-19 across a population may be associated with population mortality.

## Key Points

**Question:**

Was the sudden end of China’s zero COVID policy associated with an increase in population all-cause mortality?

**Findings:**

In this cohort study across all regions in mainland China, an estimated 1.87 million excess deaths occurred among individuals 30 years and older during the first 2 months after the end of China’s zero COVID policy. Excess deaths predominantly occurred among older individuals and were observed across all provinces in mainland China, with the exception of Tibet.

**Meaning:**

These findings suggest that the sudden lifting of the zero COVID policy in China was associated with significant increases in all-cause mortality.

## Teaser

This cohort study estimates excess mortality among adults aged 30 years or older following the end of China’s zero COVID policy.

## Introduction

During the first 3 years of the pandemic, China experienced low COVID-19–related excess mortality due to the implementation of stringent mitigation measures.^1^ However, after China ended its dynamic zero COVID policy in December 2022, COVID-19 incidence and hospitalizations surged.^2^ It has been reported by the Chinese government that approximately 60 000 COVID-19–related deaths occurred in health facilities in China from early December 2022 to January 12, 2023.^2^ Prior forecasts had anticipated a notably higher number of excess deaths if the zero COVID policy were to be abandoned during the Omicron surge, ranging from 0.97 million to 2.10 million.^3,4,5,6,7^ However, those model-based forecasts of excess deaths lacked an empirical basis.

## Methods

### Data

Mortality information was derived from published obituary data for Peking University (PKU) and Tsinghua University (THU) in Beijing and Harbin Institute of Technology (HIT) in Harbin (Heilongjiang province) from January 1, 2016, to January 31, 2023. The number of employees, including current and retired staff, of the 3 universities as of 2022 were 19 992, 19 898, and 7293, respectively. The university published the obituary of each deceased official employee on its website, with an average delay of about 3 days after the date of death. Importantly, this process occurred both before and during the COVID-19 pandemic in a consistent fashion. The obituaries were extended to all deceased official employees, regardless of their age, sex, position (eg, professor, researcher, technician, librarian, and administrative staff), and employment status (ie, currently employed or retired). Our analysis did not include employees from the affiliated hospitals of the 3 universities because their obituaries were not published on the universities’ websites.

Syndromic surveillance data were collected through search queries from Baidu, a Chinese internet search engine. The Baidu index (BI) is the weighted frequency of unique searches for a given keyword relative to the total search volume on Baidu.^8^ The number of internet users in China exceeded 1 billion as of March 2023, and Baidu search’s penetration rate reached over 90% among internet search engine users.^9,10^ The BI has been widely used as a data source for infodemiology and infoveillance studies, particularly during outbreaks of infectious disease.^11,12,13,14^ Daily BI values for mortality-related Chinese terms for “funeral parlour (殡仪馆[Bin Yi Guan]),” “cremation (火葬[Huo Zang]),” “crematorium (火葬场[Huo Zang Chang]),” and “burial (土葬[Tu Zang])” in each region (22 provinces, 4 municipalities, and 5 autonomous regions) of mainland China from January 1, 2016, to January 31, 2023, were obtained (https://index.baidu.com/v2/index.html#/). This study was exempt from institutional review approval owing to the use of published literature and publicly available data.

### Statistical Analysis

We estimated the relative change in mortality among individuals 30 years and older in Beijing and Harbin from December 2022 to January 2023 using an interrupted time-series design, a quasi-experimental design widely used to assess the causal impact of shocks or interventions introduced at a distinct point in time.^15^ We used a segmented negative binomial regression model, separating the time series into 3 periods: a pre–COVID-19 period (January 2016-December 2019), a period with stringent mitigation measures (January 2020-November 2022), and a post–zero COVID policy period (December 2022-January 2023). We included a linear association of time to capture the long-term secular trend of mortality rate. The negative binomial model equation (Equation 1) estimating monthly counts of deaths was specified as:

E(ln(Death)) = β_0_ + β_1_Month + β_2_ COVID + β_3_ ZeroCovid + offset(ln(P)).

Here, Death represents the monthly count of deaths, Month is the time (in months) since the start of the study period, COVID is an indicator variable indicating whether time occurred prior to or after the start of the COVID-19 pandemic (coded as 1 for months occurring after December 2019, and 0 otherwise), ZeroCovid is an indicator variable indicating whether time occurred prior to or after the end of the zero COVID policy (coded as 1 for months occurring after November 2022, and 0 otherwise), and P represents the catchment population (number of employees) in Month_t_. Newey-West standard errors with autocorrelation of 1 lag were used.

Similarly, we estimated the relative change in the BI associated with the lifting of the zero COVID policy in each region in China. The negative binomial model equation (Equation 2) estimating the daily BI was specified as:

E(ln(BI_i_)) = β_0_ + β_1_Day + β_2_ COVID + β_3 _ZeroCovid.

Here, BI_i_ represents the daily BI in region i, Day is the time (in days) since the start of the study period, COVID is an indicator variable indicating whether time occurred prior to or after the start of the COVID-19 pandemic (coded as 1 for days occurring after December 2019, and 0 otherwise), and ZeroCovid is an indicator variable indicating whether time occurred prior to or after the end of the zero COVID policy (coded as 1 for days occurring after November 2022, and 0 otherwise). Newey-West standard errors with autocorrelation of 1 lag were used.

**Figure 1.**
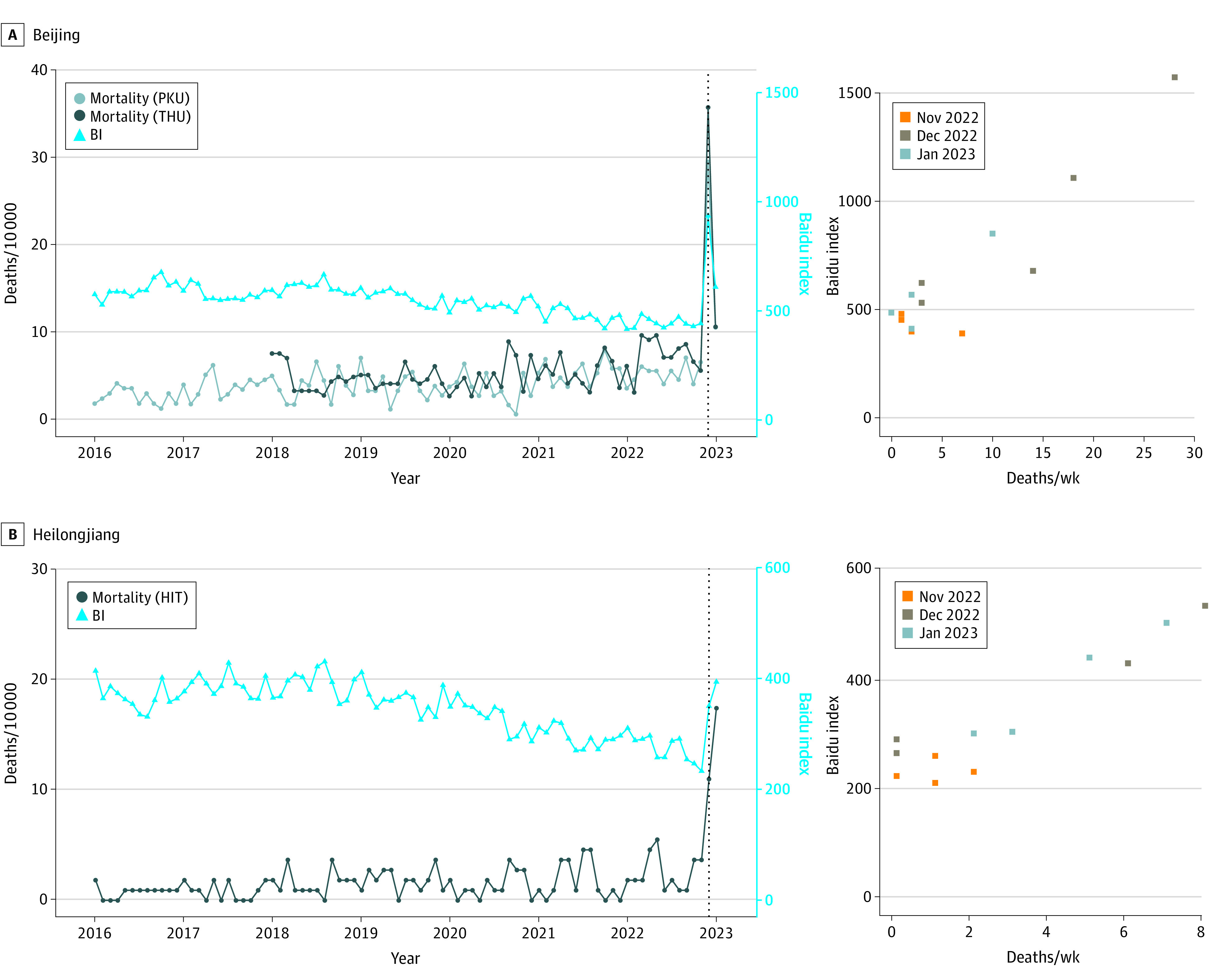
Observed Mortality and Baidu Index (BI) in Beijing and Heilongjiang Province A and B, left panels: Observed monthly mortality rate in 3 universities of China, and BI (monthly mean) in Beijing and Heilongjiang province, January 2016 to January 2023. The dotted vertical line represents the removal of the zero COVID policy in December 2022. The figure displays the BI trends, represented by light blue lines. Dark blue lines represent morality rates in Tsinghua University (THU) (A, left panel) and Harbin Institute of Technology (HIT) (B, left panel), and the light gray line represents mortality rates in Peking University (PKU) (A, left panel). A and B, right panels: Association between weekly death counts and BI (weekly mean), November 2022 to January 2023. Weekly deaths from PKU and HIT were used. Weekly death counts from THU were not obtainable. The orange, brown, and gray squares represent weeks in November 2022, December 2022, and January 2023, respectively.

We observed a strong positive correlation (Beijing: *r* = 0.95, *P* < .001; Heilongjiang: *r* = 0.97, *P* < .001) between the change in BI for mortality-related terms and that in mortality due to relaxed zero COVID policies (Figure 1). Additionally, similar patterns in the change of BI for mortality-related terms were observed in all regions of China (eFigure 1 in Supplement 1). Therefore, the relative increase in mortality in the reference region (Beijing and Heilongjiang) was extrapolated to the rest of China assuming this same proportional association (Equation 3):

(BI[RR_ref_] − 1)/(MR[RR_ref_] − 1) = (BI[RR_i_] − 1)/(MR[RR_i_] − 1).

Here, (BI[RR_ref_] − 1) and (BI[RR_i_] − 1) represent the estimated relative change in BI using Equation 2 for the reference region and region i, respectively. The estimated relative change in mortality rate for the reference region is represented by (MR[RR_ref_]-1), calculated using Equation 1, while (MR[RR_i_] − 1) denotes the projected relative change in mortality rate in region i.

Region-specific excess mortality was calculated by multiplying the proportional increase in mortality by the number of expected deaths (Equation 4):

EM_i_ = (MR[RR_i_] − 1) × Death_i_.

Here, EM_i_ is the excess mortality for region i, and Death_i_ is the expected number of deaths for region i in December and January. The expected number of deaths by region and month was derived from the 2020 census data^16^ and China National Disease Surveillance Points.^17^

Additionally, sensitivity analyses were conducted using the leave-one-out method; that is, in 3 additional analyses, we iteratively excluded 1 of the 3 universities. In both the primary and sensitivity analyses, parameter uncertainty was incorporated by randomly drawing 10 000 samples from each parameter distribution and propagating this uncertainty forward through each step of the analysis. A 2-sided *P* < .05 indicated statistical significance. Analyses were conducted in R, version 4.2.1 (R Foundation for Statistical Computing). This study is reported per the Strengthening the Reporting of Observational Studies in Epidemiology (STROBE) guidelines for cohort studies.

## Results

A total of 130 and 42 deaths occurred among employees of the 2 universities (PKU and THU) in Beijing in December 2022 and January 2023, respectively. A total of 12 and 19 deaths were reported among employees of HIT in Heilongjiang in December 2022 and January 2023, respectively. Among those deaths in Beijing, 76% (95% CI, 65%-84%) were in male individuals, and 80% (95% CI, 70%-87%) were in individuals 85 years and older, which was higher (*P* < .001) than the proportion of deaths in the 85-years-and-older age group in the prepandemic period and the first 3 years of the pandemic (Table 1). The age and sex distributions among the deaths were similar in HIT (Table 1).

**Table 1.** Observed Deaths in 3 Universities, January 1, 2016, to January 31, 2023

Characteristic	No. (%)					
	Beijing, PKU^a^			Harbin (Heilongjiang province), HIT		
	Jan 2016-Dec 2019	Jan 2020-Nov 2022	Dec 2022-Jan 2023	Jan 2016-Dec 2019	Jan 2020-Nov 2022	Dec 2022-Jan 2023
Sex						
Female	72 (26.6)	83 (29.1)	19 (24.4)	9 (22.0)	12 (20.7)	5 (17.9)
Male	199 (73.4)	202 (70.9)	59 (75.6)	32 (78.0)	46 (79.3)	23 (82.1)
Age group, y						
30-64	16 (5.4)	16 (5.2)	1 (1.2)	3 (12.5)	7 (12.3)	1 (3.4)
65-84	126 (42.7)	101 (32.6)	15 (18.8)	10 (41.6)	8 (14.0)	8 (27.5)
≥85	153 (51.9)	193 (62.3)	64 (80.0)	11 (45.8)	42 (73.6)	20 (69.0)
Title						
Academia	186 (63.1)	192 (69.8)	56 (76.7)	22 (91.7)	49 (86.0)	23 (82.1)
Administrator	91 (30.8)	62 (22.5)	16 (21.9)	2 (8.3)	6 (10.5)	3 (10.7)
Engineer/technician	18 (6.1)	21 (7.6)	1 (1.4)	0	2 (3.5)	2 (7.1)
Deaths occurred in Beijing (or Harbin for HIT)						
Yes	276 (92.9)	290 (97.6)	79 (98.8)	12 (70.6)	36 (81.8)	22 (81.5)
No	21 (7.1)	7 (2.4)	1 (1.2)	5 (29.4)	8 (18.2)	5 (18.5)
Total	297	314	80	66	71	31
Overall mortality rate (1/10 000 person-months)^b^	34.4	47.9	200.1	19.3	27.8	212.5

Abbreviations: HIT, Harbin Institute of Technology; PKU, Peking University; THU, Tsinghua University.

^a^ Demographic information derived from obituaries at THU was not obtainable. The overall death count (mortality rate) at THU during the 3 prespecified periods was 214 (23.7/10 000 person-months), 396 (57.9/10 000 person-months), and 92 (231.5/10 000 person-months), respectively. The monthly count of deaths at PKU and HIT was determined using the date of death, while the monthly count of deaths at THU was calculated based on the date that obituary was published. The median (IQR) of the difference between the date of obituary publications and the date of death was 1 (1-3), 2 (1-4), and 1 (0-3) days in PKU, THU, and HIT, respectively.

^b^ Death counts instead of mortality rate for each subgroup were used in the table because data regarding the number of employees by demographic features (eg, sex and age) were not available.

In both cities, death counts peaked in the fourth week of December 2022, concurrent with the highest BI in most provinces on December 25 (Figure 1; eFigure 1 in Supplement 1). The number of deaths in universities in Beijing showed a substantial increase compared with expected deaths, with a rise of 403% (95% CI, 351%-461%) and 56% (95% CI, 41%-73%) during December 2022 and January 2023, respectively (Table 1, Figure 1). Similarly, observed deaths in HIT were statistically significantly higher than the expected deaths both in December 2022 (12 vs 3; *P* < .001) and January 2023 (19 vs 3; *P* < .001).

The validity of our model was supported by examining whether placebo search terms (that is, search terms that are not expected to be related to the lifting of the zero COVID policy) also increased concurrently with mortality-related search terms. We found no evidence that this occurred, suggesting that mortality-related search terms through the BI served as valid surrogate for increased mortality (eFigure 2 in Supplement 1).

**Figure 2.**
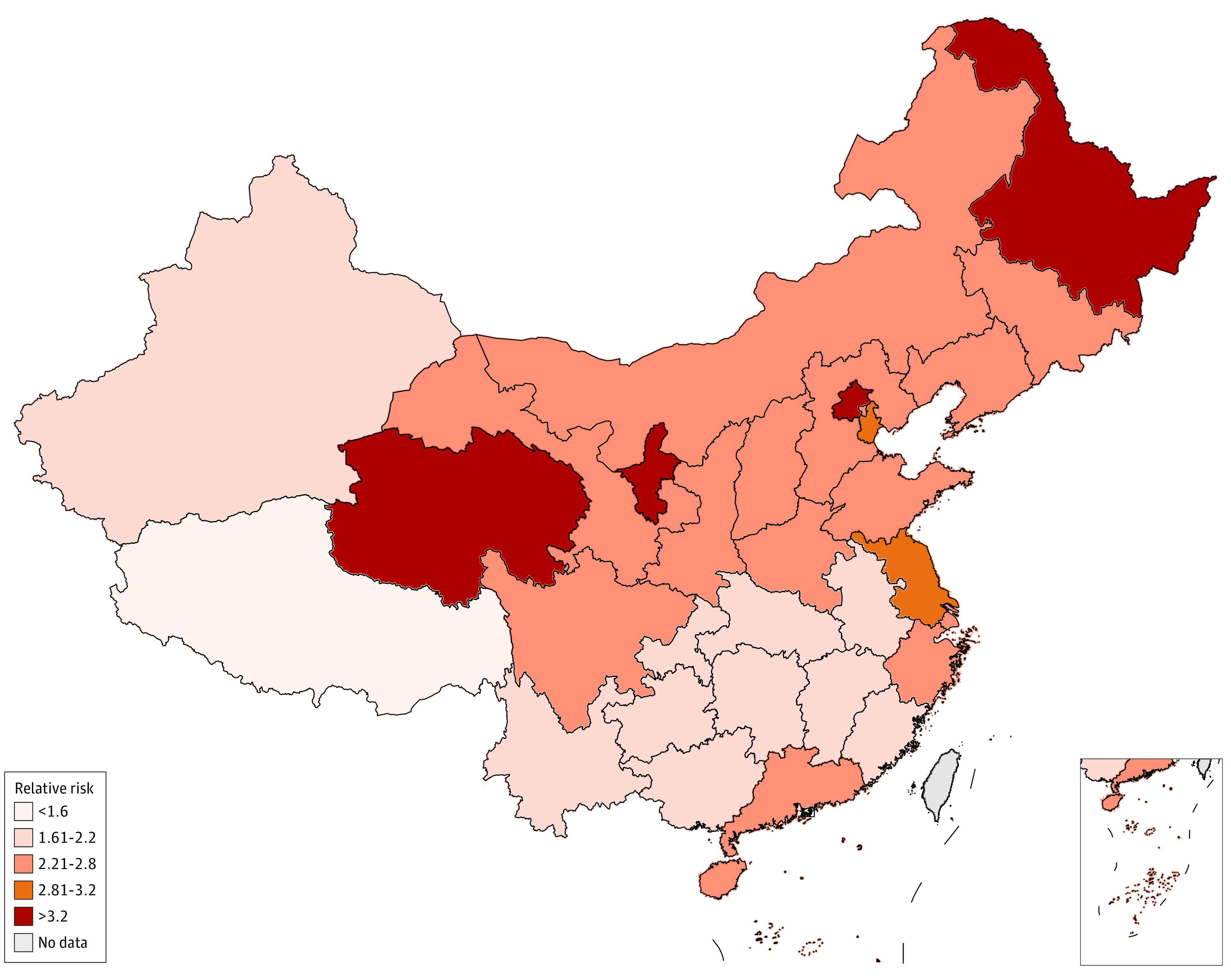
Excess Mortality Rate in China, December 2022 to January 2023

**Figure 3.**
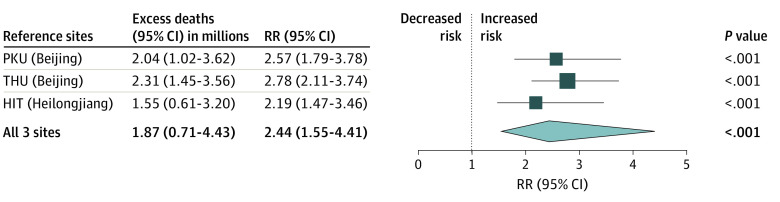
Estimates of Excess Deaths and Relative Risk (RR) of Death Using Leave-One-Out Analysis HIT indicates Harbin Institute of Technology; PKU, Peking University; THU, Tsinghua University.

Overall, an estimated 1.87 million (95% CI, 0.71 million-4.43 million; 1.33 per 1000 population) excess deaths among individuals 30 years and older occurred in China from December 2022 to January 2023. Statistically significant increases in mortality were observed in all provinces except Tibet, ranging from 77% (95% CI, 24%-197%) in Guangxi to 279% (95% CI, 109%-658%) in Ningxia (Table 2, Figure 2). Estimates for excess deaths were generally consistent in the specified leave-one-out analyses (Figure 3).

**Table 2.** Expected and Estimated Number of Deaths Among the 30-Years-and-Older Age Group by Province, December 2022 to January 2023

Region	Expected deaths^a^	Estimated deaths	Estimates (95% CI)	
			Excess deaths	Relative risk
Ningxia	5432	20 589	15 157 (5937 to 35 764)	3.79 (2.09 to 7.58)
Qinghai	3988	14 612	10 624 (3970 to 25 834)	3.66 (2.00 to 7.48)
Heilongjiang	31 377	103 943	72 566 (22 208 to 192 460)	3.31 (1.71 to 7.13)
Beijing	16 399	54 075	37 676 (15 363 to 75 570)	3.30 (1.94 to 5.61)
Tianjin	10 188	30 961	20 772 (8313 to 48 075)	3.04 (1.82 to 5.72)
Jiangsu	88 657	255 777	167 121 (64 433 to 396 887)	2.89 (1.73 to 5.48)
Inner Mongolia	20 242	56 339	36 097 (14 589 to 83 137)	2.78 (1.72 to 5.11)
Shanghai	23 276	64 240	40 964 (15 930 to 96 526)	2.76 (1.68 to 5.15)
Shanxi	24 329	67 066	42 737 (18 236 to 94 650)	2.76 (1.75 to 4.89)
Zhejiang	52 962	145 367	92 405 (36 005 to 217 513)	2.74 (1.68 to 5.11)
Henan	81 012	218 903	137 891 (58 198 to 308 011)	2.70 (1.72 to 4.80)
Liaoning	54 117	145 177	91 061 (37 007 to 208 554)	2.68 (1.68 to 4.85)
Gansu	22 315	59 380	37 065 (15 199 to 84 343)	2.66 (1.68 to 4.78)
Shaanxi	29 362	77 578	48 216 (19 730 to 109 890)	2.64 (1.67 to 4.74)
Sichuan	87 893	226 349	138 457 (54 338 to 323 803)	2.58 (1.62 to 4.68)
Hebei	67 981	170 966	102 985 (41 995 to 235 172)	2.51 (1.62 to 4.46)
Jilin	20 169	49 463	29 293 (11 179 to 69 673)	2.45 (1.55 to 4.45)
Shandong	111 414	271 773	160 359 (62 102 to 378 131)	2.44 (1.56 to 4.39)
Hainan	4646	11 100	6454 (1935 to 17 154)	2.39 (1.42 to 4.69)
Guangdong	76 284	178 278	101 994 (35 129 to 254 320)	2.34 (1.46 to 4.33)
Hubei	64 564	142 301	77 737 (28 480 to 188 212)	2.20 (1.44 to 3.92)
Xinjiang	16 369	34 957	18 588 (6225 to 47 069)	2.14 (1.38 to 3.88)
Hunan	84 770	176 992	92 222 (34 789 to 220 413)	2.09 (1.41 to 3.60)
Anhui	58 571	121 217	62 646 (23 826 to 148 957)	2.07 (1.41 to 3.54)
Chongqing	39 439	80 920	41 481 (13 957 to 104 249)	2.05 (1.35 to 3.64)
Guizhou	34 596	70 156	35 560 (13 132 to 85 919)	2.03 (1.38 to 3.48)
Fujian	35 756	71 610	35 854 (13 336 to 86 258)	2.00 (1.37 to 3.41)
Yunnan	49 040	95 586	46 545 (17 108 to 112 694)	1.95 (1.35 to 3.30)
Jiangxi	38 032	73 322	35 290 (12 138 to 87 713)	1.93 (1.32 to 3.31)
Guangxi	45 669	80 628	34 959 (10 967 to 89 765)	1.77 (1.24 to 2.97)
Tibet	1561	2478	917 (−1175 to 6242)	1.59 (0.25 to 5.00)
Total	1 300 409	3 172 102	1 871 693 (714 580 to 4 432 957)	2.44 (1.55 to 4.41)

^a^ Expected deaths by month and province had the zero COVID policy not been lifted were obtained from 2020 census data and China National Disease Surveillance Points. Data from Taiwan, Hong Kong, and Macau were not available.

## Discussion

We estimated 1.87 million excess deaths in China during the first 2 months after the end of China’s zero COVID policy. Excess deaths predominantly occurred among older individuals and were observed in all provinces in China. The number of excess deaths far exceeded official Chinese government estimates of 60 000, although the pattern of excess deaths was consistent with Chinese government reports that COVID-19–related hospitalizations and deaths in hospitals achieved its peak at the end of December 2022.^2^

The observation is consistent with prior model-based forecasts of excess deaths, trending toward a higher estimate.^5,6,18^ Airfinity’s model projected between 1.3 million and 2.1 million deaths (0.93-1.50 per 1000 population) in China if the government abruptly ended its zero COVID policy.^5,7^ Using a stochastic dynamic model of SARS-CoV-2 transmission, Cai et al^6^ projected that the Omicron wave in mainland China could cause 112.2 million symptomatic cases (79.58 per 1000 population) and 1.6 million deaths (1.10 per 1000 population), should the zero COVID policy be lifted. Drawing on the experiences of Hong Kong in 2022 as prototypes, Ioannidis et al^18^ projected 0.99 million COVID-19 deaths (0.71 per 1000 population) if the entire China population were infected after abandoning zero COVID policy. Our higher estimate than some forecasters may represent a greater effect of the SARS-CoV-2 virus on a population with limited immunity than anticipated.^3,18^

### Strengths and Limitations

Our study is among the first to provide rigorously derived, empirical estimates about excess deaths in China after the lifting of the zero COVID policy. Given the absence of comprehensive, publicly available data from China, our novel strategy for estimating excess deaths is both timely and important on this topic of public health concern both in China and internationally and demonstrates how the strategic combination of data sources can provide insights into seemingly opaque public health research questions. However, our study has limitations. The reliance on obituary data for employees from 3 universities in Beijing and Heilongjiang could result in an overestimation of excess mortality because university employees were older than the general population, or alternatively, an underestimation because the employees had higher socioeconomic status.^19^ Such biases may be especially pronounced if patterns of representation of these variables among those with obituary data changed over time. Also, increases in BI searches may not have fully reflected mortality increases outside the reference region, leading to underestimations of excess mortality in other regions. Further validation of our estimate will be crucial once alternative data sources (eg, population-based mortality data at the national or subnational level) become available. In particular, data delineated by levels of age, sex, and socioeconomic status would allow covariate adjustment for these important demographic variables.

## Conclusions

In this cohort study of the population in China, we found that the sudden lifting of zero COVID policy was associated with significant increases in all-cause mortality. Our study of excess deaths related to the lifting of the zero COVID policy in China sets an empirically derived benchmark estimate. These findings are important for understanding how the sudden propagation of COVID-19 across a population may impact population mortality.

## Notes

Supplement 1. **eFigure 1.** Daily BI by term and region (September 1, 2022-January 31, 2023) **eFigure 2.** Trends of COVID-19 related and non-COVID-19 related BI, December 2021-February 2023

Supplement 2.
Data Sharing Statement
