# Supplementary material for: Excess All-Cause Mortality in China After Ending the Zero COVID Policy
Source: JAMA Netw Open. 2023 Aug 24;6(8):e2330877. doi: 10.1001/jamanetworkopen.2023.30877 (PMC10450565; doi:10.1001/jamanetworkopen.2023.30877)
Supplement: Supplement 1. — eFigure 1. Daily BI by term and region (September 1, 2022-January 31, 2023) eFigure 2. Trends of COVID-19 related and non-COVID-19 related BI, December 2021-February 2023 [file jamanetwopen-e2330877-s001.pdf]

Supplemental Online Content

Xiao H, Wang Z, Liu F, Unger JM. Excess all-cause mortality in China after ending the zero COVID policy. *JAMA Netw Open.* 2023;6(8):e2330877. doi:10.1001/jamanetworkopen.2023.30877

eFigure 1. Daily BI by term and region (September 1, 2022-January 31, 2023)

eFigure 2. Trends of COVID-19 related and non-COVID-19 related BI, December 2021-February 2023

This supplemental material has been provided by the authors to give readers additional information about their work.

© 2023 Xiao H et al. *JAMA Network Open.*

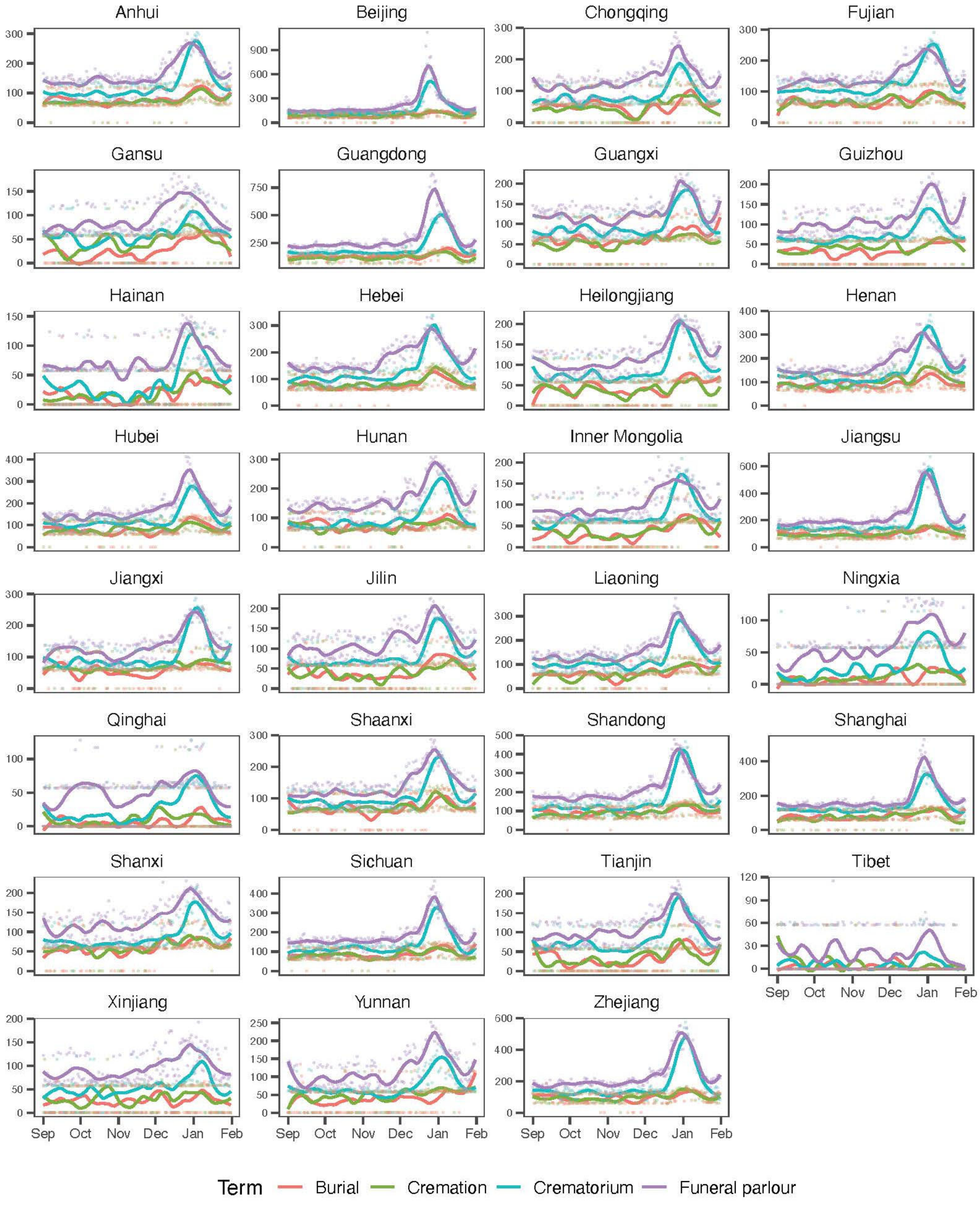

eFigure 1. Daily BI by term and region (September 1, 2022-January 31, 2023). Dots represented observed daily search index and lines indicate smoothed daily BI.

© 2023 Xiao H et al. *JAMA Network Open.*

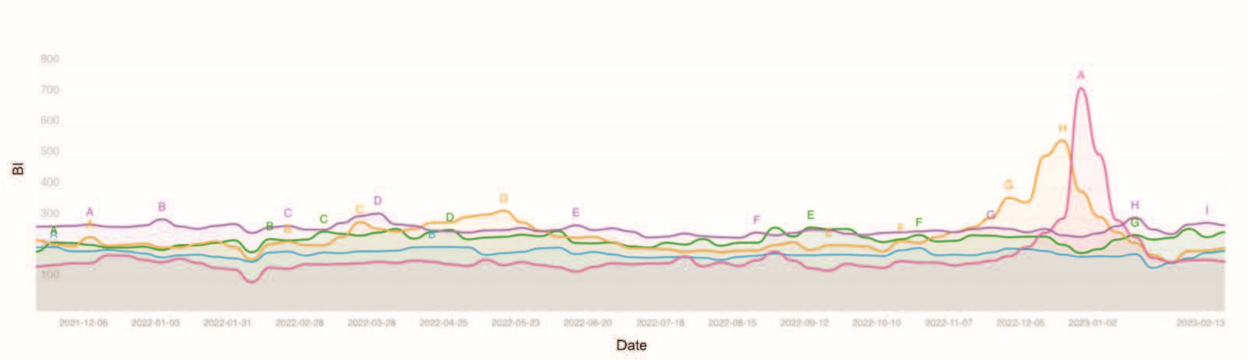

eFigure 2. Trends of COVID-19 related and non-COVID-19 related BI, December 2021-February 2023. The orange and pink line represents the BI for “mask” and “funeral parlour” respectively. The Blue, green and purple line represents the BI for “rice”, “tea” and “sunscreen” respectively. The letters denote peaks in search.

© 2023 Xiao H et al. *JAMA Network Open.*
