# Supplementary material for: Excess All-Cause Mortality in China After Ending the Zero COVID Policy
Source: JAMA Netw Open. 2023 Aug 24;6(8):e2330877. doi: 10.1001/jamanetworkopen.2023.30877 (PMC10450565; doi:10.1001/jamanetworkopen.2023.30877)
Supplement: Supplement 2. — Data Sharing Statement [file jamanetwopen-e2330877-s002.pdf]

Data Sharing Statement

Xiao. Excess All-Cause Mortality in China After Ending the Zero COVID Policy. *JAMA Netw Open*. Published August 24, 2023. doi:10.1001/jamanetworkopen.2023.30877

Data
Data available: Yes
Data types: Data (not involving human participants)
How to access data: The data sets used and analyzed during the current study are available from the corresponding authors on reasonable request.
When available: With publication

Supporting Documents
Document types: Statistical/analytic code
How to access documents: The data sets used and analyzed during the current study are available from the corresponding authors (xiaohongpku@gmail.com) on reasonable request.
When available: With publication

Additional Information
Who can access the data: Anyone requesting the data
Types of analyses: For any purpose or for a specified purpose
Mechanisms of data availability: With a signed data access agreement
